# Multidisciplinary team members’ perceptions regarding advanced psychiatric nurses’ attitudes on mental healthcare

**DOI:** 10.4102/hsag.v26i0.1646

**Published:** 2021-11-08

**Authors:** Eve P. Jacobs, Sipho W. Mkhize

**Affiliations:** 1 School of Nursing and Public Health, College of Health Sciences, University of KwaZulu-Natal, Durban, South Africa

**Keywords:** advanced psychiatric nurse, attitudes, multidisciplinary team, perceptions, psychiatric nurse, responsibilities

## Abstract

**Background:**

The attitudes of advanced psychiatric nurses significantly contribute to the management, treatment plan and care of the mental healthcare users, and resultantly affects the quality and standards of mental healthcare. Considering these effects, it is imperative to understand how the attitudes of advanced psychiatric nurses are perceived by other multidisciplinary team members.

**Aim:**

To describe the perceptions of the multidisciplinary team members regarding advanced psychiatric nurses’ attitudes in clinical practice.

**Setting:**

Three mental health institutions were utilised to obtain the perceptions related to the attitudes displayed by advanced psychiatric nurses in the clinical environment.

**Method:**

A qualitative research descriptive approach was adopted to obtain in-depth descriptions of the attitudes portrayed by advanced psychiatric nurses. Eight purposefully sampled multidisciplinary team members were interviewed to obtain information. Data were analysed using Colaizzi’s method.

**Results:**

Themes and sub-themes emerged. Mental healthcare providers exhibited both negative and positive attitudes towards mental health patients. By perception, unfavorable attitudes hindered effective communication and psychosocial rehabilitation programs for mental health patients. Positivity enhanced feedback during ward rounds and during clinical meetings.

**Conclusion:**

Despite the positive impact advanced psychiatric nurses have on mental health care, views regarding mental health patients remain negative. It is recommended that advanced psychiatric nursing education curriculum address negative attitudes, views, and stereotypes. Mental healthcare users need to be provided with psychosocial rehabilitation programs and activities that address advance psychiatric nurses’ lack of initiatives.

**Contribution:**

A clear set of principles and protocols underpins the collaborative effort among multidisciplinary teams in mental health care.

## Introduction

The multidisciplinary team (psychiatrists, advanced psychiatric nurses, psychologists, occupational therapists, social workers) are essential for the care of mental healthcare users, considering the biological, psychological and social aspects. Psychiatric nurses are recognised as vital contributors to the care and treatment of the mentally ill, which determines the extent and level of quality care given to the mental healthcare users (Al-Awadhiet al. 2017:32; Kneisl & Trigoboff 2013:26; Uys & Middleton 2014:39). It is therefore important to obtain the views of the multidisciplinary team about the observations made on the attitudes of the advanced mental health nurses in mental healthcare.

The dire need to improve the status of mental healthcare and service delivery, according to Schober (2016:49), stimulated the implementation of advanced psychiatric competencies in South Africa through the training of advanced psychiatric nurses. Martensson, Jacobsson and Engstrom (2014:782) concluded that mental health training and knowledge contribute to positive attitudes that have an impact on strengthening the mental healthcare provider’s attitudes towards mental healthcare users. They summarised that whilst mental health illness training and knowledge contributed to positive attitudes, organisations had a great influence contributing towards the general attitude of the staff towards the mental healthcare users.

In this article, a brief definition of attitudes, perceptions and their components are described. Morvan and O’Connor (2017:1) and Openstax (2019:81) stated that an ‘attitude’ was the susceptibility to respond in a manner that would be favourable or unfavourable to individuals in their given setting. There are three components interrelated to attitudes. The *cognitive component* focuses on beliefs and ideas of individuals concerning people, substances, and environments around them. The *affective component* is related to how individuals manage their feelings towards people, objects, and environments. The *intentional component* relates to individuals’ behavioural intentions towards the people or environment involved.

In defining ‘perception’, Qiong (2017:18) mentioned that it included one’s natural ability to make sense and gain understanding of certain thoughts towards something and how it is interpreted. Perceptions in this study refers to how the advanced nurses embrace and utilise their knowledge to care for the mental healthcare users effectively.

In understanding the concept of nursing attitudes and their effects on nursing practice, Price (2015:50) mentioned that attitudes formed a critical component in assisting nurses to determine what they deemed as significant, virtuous, suitable and imperative. Moreover, nurses would possess the ability to ensure the provision of a more collaborative and patient-centred approach. According to Tambag (2018:420), individual negative attitudes, beliefs and ideas have an impact on the social recognition and treatment of mental healthcare users, which can be the causative factor in the impairment of mental healthcare users’ treatment and care results.

### Problem statement

According to Joubert and Bhagwan (2018:54) and Setona, Sehularo and Makgaola (2020:2), advanced psychiatric nurses face many challenges that impede the effective utilisation of their speciality in South Africa. Basson, Julie and Adejumo (2014:3) stated that with the increasing demand in mental illness, the general attitude of professional nurses, their level of work experience and their training would determine whether they display negative or positive attitudes. Based on the clinical experience of the researcher and collaboration with the multidisciplinary team, the attitudes of the advanced psychiatric nurses observed were still negative, despite their training. Moreover, mental healthcare in the study context remained unchanged because the treatment remained custodial. Therefore, there is a need to understand the factors contributing to the negative attitudes of advanced psychiatric nurses, ways to promote positive attitudes and to establish whether psychiatric nursing training has any impact on mental healthcare. There is minimal information about advanced psychiatric nurses’ nursing attitudes in South Africa in the clinical area after education and training. It is unknown what effect advanced psychiatric nurses’ training has on the attitudes of advanced psychiatric nurses towards mental illness and treatment of the mental healthcare users. There is no information related to changes in their attitudes and to what extent they affect the treatment plans and care of the mental healthcare users.

### Purpose

The purpose of this article is to describe the perceptions of the multidisciplinary team members regarding the advanced psychiatric nurses’ attitudes towards mental healthcare.

### Objective

The objective of this article is to describe the perceptions of multidisciplinary team members regarding advanced psychiatric nurses’ attitudes towards mental healthcare.

## Research methodology

According to Kim, Sefcik and Bradway (2017:1), studies that require examination and description in natural contexts adopt qualitative description. This study used a qualitative approach with an exploratory descriptive design as it focused on discovering and gaining insights about advanced psychiatric nurses’ attitudes towards mental healthcare.

### Study design

The researcher adopted a qualitative approach with a descriptive design. The participants described their perceptions about the attitudes of advanced psychiatric nurses towards mental healthcare and treatment of mental healthcare users. Individual interviews were utilised in the naturalistic setting to attain in-depth information on their perceptions of advanced psychiatric nurses’ attitudes towards mental illness.

### Study setting

The study took place in uMgungundlovu district, KwaZulu Natal province, with the sampling of three specialised mental health institutions.

### Study population and sampling

Majid (2018:3) referred to population as the targeted population of interest that is intended to be studied. The population in this study were psychiatrists, psychologists and medical officers employed and with work experience in the specialised mental health institutions. Eight participants, comprising three psychologists, two medical officers and three psychiatrists, who worked on a rotational basis at the three specialised mental health institutions, received an invitation for the face-to-face interviews. Purposeful sampling was utilised as the participants, who were regarded as experts, could provide valuable information in the field under study (Elo et al. 2014:4). The eligibility criteria in this study required participants employed within the psychiatric institution for a period of four years and above, and having experience of working with advanced psychiatric nurses and the mental healthcare users. The participants contributed rich information relating to the purpose of the study and had the knowledge and experience of having worked with advanced psychiatric nurses over a specified period. Therefore, they could describe the attitudes displayed by advanced psychiatric nurses in clinical practice.

### Data collection

The process of data collection commenced immediately after ethical clearance was received from the Biomedical Research Committee of the University of KwaZulu Natal, Department of Health, and from the chief executive managers and nurse managers of the institutions recruited for the study. The data collection process included eight participants in the one-to-one interviews, conducted until the reaching of data saturation, which lasted for three months, from July 2019 to September 2019. The researcher commenced the interviews by using the broad question: *During your years of experience and since the inception of the advanced psychiatric nurse’s role, what are your perceptions of advanced psychiatric nurses’ attitudes towards mental illnes*s? The researcher probed further to obtain a deeper understanding of the perceptions shared and asked the following question: *From the attitudes displayed by the advanced psychiatric nurses how has this had an impact in caring for the mental healthcare users?* The participants were asked to provide explanations to the researcher in areas that needed more clarity.

### Data analysis

Colaizzi’s method of data analysis, as described by Kusi et al. (2020:5), was used to analyse the data in this qualitative study. All the interviews were audio-recorded and transcribed verbatim. The field notes corroborated with the audio-recorded information during open coding process. The researcher used the pseudonyms allocated to the participants during the transcription process of. The eight transcripts were read repeatedly to make sense of the shared perceptions on the attitudes displayed by advanced psychiatric nurses. The significant recurring statements and phrases to describe the attitudes of the advanced psychiatric nurses were extracted from the transcripts and thereafter coded. Formulated meanings were created from the significant statements and phrases to describe the meanings. The themes generated arose from the statements that conveyed similar meanings. The researcher then categorised all the formulated meanings into a cluster of themes. An exhaustive description was created and the findings were summarised into rich descriptions of attitudes displayed by advanced psychiatric nurses in the clinical setting. This also provided an opportunity for additional or new information to be gathered.

### Measures to ensure trustworthiness

To ensure authenticity of the study findings, the four strategies of trustworthiness namely, credibility, dependability, confirmability and transferability were employed (Nyirende et al. 2020:2). Credibility ensured that the findings obtained were congruent through prolonged engagement and member checking. Dependability ensured there was sufficient information given so that there could be replication of the study. Confirmability was achieved by the researcher by being unbiased throughout the study and by providing clear descriptions to ensure that all the shared information of the participants was a true reflection of what was shared. Transferability was achieved as the researcher included a clear guide on the contexts followed throughout the study. The researcher ensured trustworthiness by making use of one-to-one interviews to collect data from the willing participants. The researcher used a reflective diary in combination with the interviews (Elo et al. 2014:3).

### Ethical considerations

Ethical clearance was obtained from the University of KwaZulu-Natal Biomedical Research Ethics Committee (BREC reference number: 362/18). The Gatekeeper KwaZulu Natal, Department of Health Research Ethics Committee (NHRD reference: KZ_201808_20) granted permission for this study to proceed. According to Arifin (2018:30), the protection and safety of the participants need to be ensured by adhering to the following ethical principles: informed consent, anonymity and confidentiality. The participants freely volunteered themselves and gave written consent for the interviews after they received full information about the study, including the fact that they could withdraw at any time without any effect on them. The use of pseudonyms replaced participants’ names to protect them; they had to use their pseudonyms during the audio-recordings. The individual interviews were conducted separately in a quiet room to ensure confidentiality.

## Findings

Table 1 illustrates the demographic data of the participants included in the one-to-one interviews, including their gender, age and years of experience.

**TABLE 1:** Demographic data.

Age in groups	Number (*N*)	Females	Males	Years of experience
Psychologists				
36–45	1	0	1	10
46–55	1	0	1	20
56–61+	1	1	0	30+
Psychiatrists				
46–55	2	2	0	30+
56–61+	1	1	0	35+
Medical officers				
36–45	2	1	1	15

Two themes and four sub-themes emerged from the data analysis and they are described below:

### Theme 1: Advanced psychiatric nurses’ negative attitudes towards mental healthcare users

The participants indicated that the advanced psychiatric nurses displayed negative attitudes. The negative attitudes included ineffective communication, speaking harshly, displaying verbal aggression and raising their tone of voice by screaming at the mental healthcare users. These negative attitudes lead to poor facilitation or implementation of psychosocial rehabilitation programmes.

#### Subtheme 1.1: Lack of interest to initiate psychosocial rehabilitation programmes

The participants reported that the advanced psychiatric nurses demonstrated limited interest in initiating or facilitating psychosocial rehabilitation programmes for mental healthcare users in the clinical setting. There were limited activities carried out as part of rehabilitating the mental healthcare users. Some of the participants were of the view that the contributory factor could be the type of mental illness of the mental healthcare users as a causative factor in hampering their participation in psychosocial rehabilitation programmes. The participants echoed that advanced psychiatric nurses were capable of driving psychosocial rehabilitation programmes, yet did not exploit their capabilities. The participants provided the following quotes to indicate the need for advanced psychiatric nurses to participate and facilitate psychosocial rehabilitation programmes:

‘The advanced psyche nurses actually need to devise or adapted a PSR for children and adolescents, and they take the information, or they fill in that form, and they have to adapt according to age, cognitive ability.’ (Psychiatrist 3, female, 60 yrs old)

‘So most of our patients cannot be rehabilitated. Uhm…to an extent that they can be discharged… and they need to do some activities and help them where they need help.’ (Medical officer 2, female, 36 yrs old)

‘In terms of psychosocial rehabilitation, I think both. Because like I said, in the community, there isn’t anyone that has that experience. Psychosocial rehab is long-term. So, in terms of in-patient, I would think their role would be in starting this therapy, dealing with patients that aren’t that stable.’ (Psychiatrist 1, female, 52 yrs old)

‘I don’t think anybody is doing psychosocial rehabilitation (laugh) that is an odd question to answer. But yes, I think that the advance…. the nurse should be at the forefront of PSR.’ **(**Psychiatrist 2, female, 55 yrs old)

‘Psychosocial rehab is very important and she needs to drive it down there so that we prevent revolving door syndrome.’ (Psychiatrist 2, female, 46 yrs old)

The lack of interest to initiate psychosocial rehabilitation programmes reflected the concerns verbalised by the participants, which links with the following sub-theme.

#### Subtheme 1.2: Ineffective communication skills when dealing with mental healthcare users

In this study, ineffective communication refers to the inability of the advanced psychiatric nurses to respectfully speak with the mental healthcare users. The participants observed that the advanced psychiatric nurses would sometimes speak in a condescending manner when communicating with the mental healthcare users. The participants felt that the advanced psychiatric nurses should establish a good rapport with mental healthcare users and try to gain their trust as they communicate with them. They should speak calmly and show good listening skills, empathy and understanding when dealing with the mental healthcare users to establish an effective therapeutic relationship.

‘They’re could do much better at maintaining rapport with the patient which is helpful in therapy.’ (Psychiatrist 1, female, 52 yrs old)

‘They lack confidence…So it’s also about communication skills, about empathy, besides just the clinical knowledge, and also, just human understanding.’ **(**Psychologist 3, male, 40 yrs old)

‘You’d be seeing them calling them names and grabbing them and you’d be like, this person is not empathetic and maybe they are not made for psychiatry. You have to love psychiatry before you can practice in the psyche because this can be emotionally draining.’ (Medical officer 2, female, 36 yrs old)

The sub-theme stated above revealed the existence of some of the negative attitudes that still affect mental healthcare. Realisation of gaps and causative factors and educative intervention strategies could lead to more positive attitudes being adopted in future.

### Theme 2. Advanced psychiatric nurses’ positive attitudes towards mental healthcare users

The participants indicated that the positive attitudes of advanced psychiatric nurses were reflected through implementing appropriate competencies acquired in training as part of the multidisciplinary team. These competencies enhanced their ability to manage the mental healthcare users effectively. It was evident that advanced psychiatric nurses were fully cognisant of their role expectations in providing comprehensive feedback regarding the condition and progress of the mental healthcare users.

#### Subtheme 2.1: Competency levels

The participants stated that the competency levels of advanced psychiatric nurses stood out in caring for mental healthcare users of various age groups. The ability to function independently showed through their presentation skills and strategies in managing various forms of mental health disorders across levels of care and treatment. The number of years of experience in the mental healthcare environment strengthened the competency levels that were initially acquired at the education and training setting.

‘Diploma or a degree that you qualify for, but when you get that certificate, it must mean that you have gained certain competencies as an Advance Mental Healthcare Nurse to be able to even work independent.’ (Psychiatrist 3, female, 60 yrs old)

‘…They have done the course in terms of mental health service, they have passed the course in advanced psychiatry and would be very offay with common psych disorders, management plans that are in place.’ (Psychiatrist 1, female, 52 yrs old)

‘Undergo further qualification and study into an advanced psych, they are advanced mental health practitioner. So, are highly qualified nursing practitioner and are skilled within mental health practice.’ (Psychiatrist 2, female, 46 yrs old)

#### Subtheme 2.2: Comprehensive information and feedback during ward rounds

The participants reported that advanced psychiatric nurses’ knowledge and understanding of the mental healthcare users were more comprehensive than a nurse who had not been trained. They displayed efficiency during ward rounds or clinical meetings by providing adequate feedback regarding the mental healthcare users’ conditions. Advanced psychiatric nurses’ ability to integrate theory to practice combined with their years of experience contributed to detailed and specific feedback.

‘Uhm … I feel that they do come with a better understanding of the patient, they do understand more when we’re … it’s very helpful to have them in ward rounds because when we’re asking certain questions that your younger nurses might not understand what we are trying to elicit information from, they understand exactly the type of information we are wanting to get out of the patient.’ (Psychiatrist 1, female, 52 yrs old)

‘The Advance Nurses are the more senior nurses in terms of experience and also then have a certain level of qualifications. And they contribute greatly to one’s understanding of patients. They able to offer rich knowledge.’ (Psychologist 1, male, 61 yrs old)

‘Some of the nurses that I have worked with for 30, 35 years has the experience and uh… it is quite clear that they can integrate that, during ward rounds.’ (Psychologist 2, female, 55 yrs old)

‘When we have a weekly clinical meeting where we discuss all the patients, each discipline has a chance to explain, and the nurses who are there with them 24/7 have a huge role to play. Uhm … so they also present. Uhm … part of their information, giving information thoroughly to the team.’ (Psychologist 3, male, 40 yrs old)

## Discussion of findings

The multidisciplinary team members who formed part of the study identified that the advanced psychiatric nurses displayed both negative and positive attitudes towards mental healthcare. They provided explanations of how negative attitudes were displayed in their communication and how the lack of psychosocial rehabilitation affected mental healthcare. Simultaneously, they acknowledged and commended the advanced psychiatric nurses for displaying positive attitudes in using skills to provide comprehensive feedback to the multidisciplinary team regarding the mental healthcare users.

### Theme 1: Advanced psychiatric nurses’ negative attitudes towards mental healthcare users

In the study, the negative attitudes of the advanced psychiatric nurses are reflected in their ineffective communication skills while interacting with the mental healthcare users. This has resulted in a lack of rehabilitation for the mental healthcare users, as there are no psychosocial rehabilitation programmes facilitated by the advanced psychiatric nurses. Communication and psychosocial rehabilitation are entities that are viewed as being fundamental in mental healthcare (Saha, Chauhan & Pandya 2020:893).

According to the study conducted in Sweden, Martensson et al. (2014:782) revealed that psychiatric nurses display negative attitudes. Martensson et al. (2014:782) alluded that the contributing factor was the lack of relevant knowledge and skills. This is contrary to the South African Nursing Council’s (SANC) competencies, which stipulate that once trained, the advanced psychiatric nurses should display good communication skills and therapeutic relationships (SANC 2019:3).

According to Mullen (2009:85), Wardani (2014:644) and SANC (2019:1), psychiatric nurses are in a good place and able to provide psychosocial rehabilitation as they are constantly dealing with the mental healthcare users. An earlier study by Mullen (2009:85) indicated that psychosocial rehabilitation was an agreed function for even the basic psychiatric nurse. However, the author identified challenges that hinder the psychosocial rehabilitation interventions, such as acute mental illness, psychotic behaviour, repeated abscondment and use of bio-medical interventions. This concurs with Parker (2012:418), who implied that the bio-medical model remains the focal point for symptom reduction in comparison to treatment approach focussing on adoption of a full functional integration into society. Wardani (2014:644) was of the view that the advanced psychiatric nurses were fearful to initiate psychosocial rehabilitation programmes because of the aggressive behaviours displayed by some mental healthcare users. According to Saha et al. (2020:892), routine use of psychosocial rehabilitation programmes is a critical aspect of mental healthcare programmes. Psychosocial rehabilitation provided a solution to the chaotic state of the inpatient institutions and promoted treatments that would ensure high functional living for the mental healthcare users in society (Saha et al. 2020:892). The South African Psychosocial Rehabilitation policy, introduced in 2010, was implemented to offer the opportunity for mental healthcare users to reach high levels of individual and independent functioning in the community (Botha et al. 2020:2; DOH 2010:2). Psychosocial rehabilitation interventions promote mental well-being, healthy lifestyle choices, autonomous living and a reduction in hospital admissions (Rasmus et al. 2021:2).

Baziga (2013:6) stated that the attitudes of nurses have a role to play in mental healthcare users’ treatment initiation and the course it would take. After all, the SANC identified that advanced psychiatric nurses should be nurse specialists, competent and able to provide the prescribed psychosocial rehabilitation services (SANC 2019:1). Additionally, they needed to apply their clinical skills to develop and conduct recovery-based psychosocial rehabilitation approaches.

Communication forms an integral part of therapeutic interventions in mental health nursing (Furnes, Kvaal & Hoye 2018:1). As a result, the communication approach used for mental healthcare users requires empathy, accountability and sensitivity. Communication provides benefits of problem solving for the mental healthcare users, and further facilitates the development of a positive nurse-patient relationship (Ali 2018:1; Furnes et al. 2018:3; Morrissey & Callaghan 2011:1). Thus, advanced psychiatric nurses should display rapport, empathy and observation skills effectively to be confident in dealing with mental healthcare users. The SANC’ core competencies (SANC 2019:12) affirm that the advanced psychiatric nurses should competently establish and maintain rapport. Yet despite this, the study has revealed that some advanced psychiatric nurses lack poise in effective communication when dealing with the mental healthcare users.

The advanced psychiatric nurses reportedly did not show empathic responses in their attitudes when they handled mental healthcare users. According to Uys and Middleton (2014:498), psychiatric nurses should not be raising their voices when communicating with mental healthcare users. According to the SANC (2019:12), responsibilities of advanced psychiatric nurses are to establish and maintain therapeutic relationships through communication. Study findings from Sim, Ahn and Hwang (2020:10) revealed that advanced psychiatric nurses displayed indifference instead of empathy towards mental healthcare users. Unruly aggressive and violent behaviours of the mental healthcare users were apparently contributory factors in provoking lack of empathy, consequently leading to verbal aggression displayed towards them. Similarly, an earlier study by Egbe et al. (2014:5) revealed that advanced mental health nurses verbally abused mental healthcare users in healthcare facilities. A recent African study conducted by Sahile et al. (2019:6) revealed that professional nurses, without or with basic psychiatry nursing, displayed more negative attitudes towards mental healthcare compared to psychiatric nurses who had completed advanced training.

The authors were of the view that there should cognisance taken to determine additional factors on how to reduce negative attitudes and perceptions towards mental healthcare.

### Theme 2: Advanced psychiatric nurse’s positive attitudes towards mental healthcare users

Uys and Middleton (2014:169) stated that a positive attitude displayed by a psychiatric nurse communicated caring for mental health care users through an empathic approach. This means psychiatric nurses are required to be empathetic in understanding mental healthcare users’ conditions and behaviours.

Therefore, to display positive attitudes, advanced psychiatric nurses should be educated and trained to carry out their role functions. Martensson et al. (2014:783) concurred that psychiatric nurses, who were frequently in contact with the mental healthcare users, displayed positive attitudes towards them. According to Sahile et al. (2019:6), increased personal contact with mental healthcare users reduced psychiatric nurses’ negative attitudes. The study by Basson et al. (2014:524) affirmed that advanced psychiatric nurses displayed more positive attitudes than the professional nurses with basic psychiatric nursing training.

De Kock and Pillay (2016:2) revealed that socio-economic risk factors in South Africa, as a middle-income country, contributed to an increased risk of intergenerational mental illness. Sehularo (2016:1) identified the shortage of specialised psychiatric nurses in South Africa as a deficiency in advanced psychiatric nursing competencies, which impede management, and treatment of mental healthcare users. Support in the education and training of advanced nurses critical thinking and clinical reasoning skills would promote mental health treatment (Sehularo 2016:1).

Advanced psychiatric nurses have an important role during ward rounds as they share information between the multidisciplinary team and the mental healthcare users (Royal College of Physicians, Royal College of Nursing 2012:3; Zamazadeh et al. 2021:97). The advanced psychiatric nurses’ responsibility is to undertake the ward rounds and ensure smooth facilitation to be able to provide feedback to other staff members (Lees 2013:12). Literature reveals that ward rounds in the multidisciplinary team convey information, provide opportunities for effective communication between mental healthcare users and the multidisciplinary team, and enhances collaborative decision-making (Mattinson & Cheeseman 2018:1; Vietz et al. 2019:1). Mattinson and Cheeseman (2018:1) indicated that ward rounds, provided sufficient opportunities for the development and review of weekly mental healthcare plans and mental healthcare users’ treatment progress.

## Conclusion

Despite the influence that advanced psychiatric nurses have on mental health treatment and care, there are negative attitudes displayed towards mental healthcare users. The recommendation is for a strategic review of the nursing curriculum that addresses negative attitudes, perceptions and stereotypes, especially at the advanced training level. The advanced psychiatric nurses’ lack of initiative to implement psychosocial rehabilitation programmes and activities needs addressing to improve psychosocial rehabilitation for mental healthcare users. It is essential that advanced psychiatric nurses are equipped with effective skills to communicate therapeutically and initiate psychosocial rehabilitation programmes to enhance quality and functionality in mental health.

### Recommendations

The advanced psychiatric nursing curriculum needs to be strategically reviewed to address negative attitudes and perceptions that still affect advanced psychiatric nurses whilst in the clinical setting. Studies on advanced psychiatric nurses’ attitudes and perceptions towards mental health is scarce worldwide, including South Africa. Several studies focused on professional nurses with basic psychiatric nursing training to find out their attitudes and perceptions towards mental illness. Therefore, more studies need to be conducted to discover attitudes of advanced psychiatric nurses within clinical practice to ascertain if the advanced training had any influence on their attitudes, what attitudes they have adopted, and how to best facilitate these to strengthen the role fulfilment of the advanced psychiatric nurses.

### Limitations

The literature support for this study was mostly from findings of international studies. South Africa has minimal literature that discusses the attitudes and perceptions of advanced psychiatric nurses in clinical settings. Therefore, the findings cannot be generalised to the advanced psychiatric nurses in the South African context. The study was limited to uMgungundlovu district, with the other two districts in the province not included.
